# Probing the Depth-Resolved Structure of Adsorbed Azobenzene Surfactant Films by QCM‑D

**DOI:** 10.1021/acs.langmuir.6c00926

**Published:** 2026-06-03

**Authors:** Maren Umlandt, Philipp Ortner, Nino Lomadze, Marek Bekir, Svetlana Santer

**Affiliations:** Institute of Physics and Astronomy, 26583 University of Potsdam, Potsdam 14476, Germany

## Abstract

Adsorbed films of photoswitchable surfactants exhibit complex internal organization that critically determines their interfacial mechanics and functionality. Here, we use quartz crystal microbalance with dissipation monitoring (QCM-D) to resolve the depth-dependent structure of azobenzene-based surfactant layers adsorbed at borosilicate glass–water interfaces. Analysis of frequency and dissipation responses across multiple overtones allows us to distinguish contributions from film regions that are rigidly coupled to the oscillatory shear field from those that remain weakly bound and are highly hydrated. We observed a pronounced concentration-dependent reorganization of the adsorbed layer. Below the critical micelle concentration (CMC), adsorption results in compact, mechanically rigid mono- to bilayer structures formed by tightly packed surfactant molecules. Above the CMC, excess surfactant accumulates in an extended, water-rich overlayer that contributes strongly to dissipation at low overtones but only marginally to the shear-coupled mechanically active film thickness. These results provide quantitative insight into the vertical viscoelastic profile of azobenzene surfactant films and demonstrate the capability of QCM-D to resolve the internal structural heterogeneity in responsive interfacial systems.

## Graphical abstract

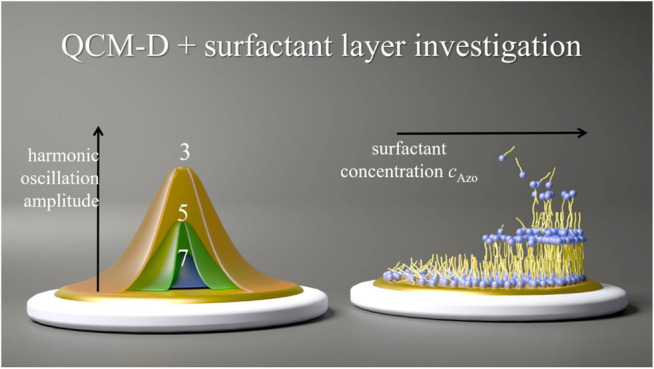

## Introduction

A detailed understanding of amphiphile adsorption and interfacial behavior at solid–liquid interfaces is critical for elucidating diverse physicochemical phenomena and optimizing applications ranging from detergency and coatings to nanoparticle functionalization, environmental remediation, lubrication, and pharmaceutical formulations.^1–7^ Despite extensive studies, resolving how adsorbed surfactant layers evolve dynamically, particularly at low coverage or in response to external stimuli, remains a significant challenge. Conventional techniques often provide only equilibrium snapshots, lacking the lateral or temporal resolution required to capture the formation and evolution of partially covered or heterogeneous layers.^8–10^

The nature of the interactions between surfactant molecules and the substrate, including electrostatic attraction, hydrogen bonding, covalent bonding, and hydrophobic forces, critically determines the structure and morphology of the adsorbed layer. On hydrophobic surfaces, adsorption typically produces sparsely adsorbed monolayers resembling the air–solution interface,^11^ whereas on hydrophilic surfaces, adsorption is often cooperative, leading to surface-associated aggregates.^12,13^ Beyond simple mono- and bilayer formation, various aggregate morphologies have been reported, including adsorbed hemimicelles (monomers adsorbed with headgroups at the surface and hydrocarbon chains extending into the solution),^12–14^ spherical admicelles (headgroups oriented toward both the substrate and the solution with hydrophobic cores),^15–17^ hemicylindrical aggregates, and fully or partially cylindrical structures, particularly on mica, glass, silica, or graphite interfaces.^18,19^

Most of the current understanding of these structures comes from studies of conventional ionic surfactants adsorbing on model hydrophilic substrates. For example, cationic quaternary alkyl ammonium bromides (C_n_TAB, n = 8–20), such as cetyltrimethylammonium bromide (C_16_TAB), have been extensively studied on mica, amorphous silica, and thermally oxidized silicon or quartz using AFM,^8–10,20,21^ SFA,^22,23^ neutron^24^ and X-ray reflectivity,^25,26^ X-ray photoelectron spectroscopy,^27^ and simulations.^28^ These studies consistently show that at concentrations above the critical micelle concentration (CMC, ∼0.9 mM at 25 °C),^29,30^ and under thermodynamic equilibrium, the mica surface is completely covered by a C_16_TAB bilayer (∼3.2 nm thickness), indicating tilted or interdigitated molecular packing, as the expected fully extended bilayer thickness would be ∼4.7 nm.^26^ While these studies provide detailed insight at full surface coverage,^31^ they offer limited information on partially covered surfaces or the dynamic evolution of adsorption, especially for functional or stimuli-responsive surfactants.

Techniques such as AFM or neutron/X-ray reflectometry, although capable of high-resolution measurements, are limited in providing lateral and temporal information and often require assumptions or complex experimental setups.^32–36^ In contrast, quartz crystal microbalance with dissipation monitoring (QCM-D) provides a versatile, label-free approach to simultaneously quantify adsorbed mass via frequency shifts and assess viscoelastic properties through energy dissipation measurements.^37–41^ QCM-D can operate under diverse environmental conditions (liquid, gas, vacuum) and on substrates of varying roughness, enabling real-time, in situ monitoring of structural and conformational changes in adsorbed molecular layers at solid–liquid interfaces.^42–46^

In this study, we focus on the adsorption of a photoresponsive azobenzene-based cationic surfactant at a negatively charged borosilicate glass–water interface, where light-responsive molecular conformations introduce additional complexity to the adsorption process. Azobenzene-containing surfactants were selected because they belong to a class of photoresponsive amphiphilics capable of reversible trans–cis isomerization under light irradiation. This molecular switching can induce pronounced changes in surfactant packing, interfacial organization, and viscoelastic properties at solid–liquid interfaces. Establishing the adsorption behavior of the trans-rich state therefore provides an important reference for future studies of light-controlled interfacial restructuring and related photoresponsive transport phenomena. We employ QCM-D to investigate how the adsorbed layer evolves from a low-coverage rigid monolayer to a diffuse, hydrated multilayer as the surfactant concentration increases, with the multilayer forming immediately upon exceeding the CMC. The structural interpretation relies on the frequency-to-dissipation ratio across multiple overtones, where lower harmonics probe deeper into the bulk solution compared with higher harmonics.^47–50^

Previous studies investigated the adsorption behavior of AzoC_6_ at borosilicate surfaces using conventional QCM-D analysis. However, these approaches primarily provided information about the total adsorbed mass.^51,52^ The present work extends this analysis by employing overtone-resolved QCM-D measurements and viscoelastic modeling to resolve the depth-dependent mechanical structure of the adsorbed surfactant layer.

Importantly, our study addresses a key gap in the current understanding of surfactant adsorption at solid–liquid interfaces. While previous investigations have characterized fully covered layers of conventional ionic surfactants,^15–26^ the structure and dynamics of partially covered or low-density layers, especially for stimuli-responsive surfactants, remain largely unexplored. By combining overtone-resolved QCM-D with viscoelastic modeling, we provide real-time, depth-resolved insight into the mechanical and structural evolution of a photoresponsive azobenzene-based cationic surfactant layer on borosilicate glass. This approach allows us to capture both mass adsorption and layer viscoelasticity simultaneously, revealing dynamic processes that are inaccessible to conventional techniques such as AFM or reflectometry.^15,32–36^

## Materials

Azobenzene-containing surfactant 6-[4-(4-Hexylphenylazo)-phenoxy]-butyl-trimethylammonium bromide (C_4_-Azo–O-C_6_TMAB, here abbreviated as **AzoC**_**6**_) is synthesized following a previously reported procedure.^53^ The identity and purity of the final product were confirmed by ^1^H NMR spectroscopy (see Supporting Information Figure S1). The molecule comprises a trimethylammonium bromide headgroup connected to an azobenzene moiety via a spacer of six methylene units, and a terminal butyl group attached to the aromatic unit (Figure 1a). The critical micelle concentration of *trans*-AzoC_6_ under the present conditions is approximately 0.5 mM (∼1 × CMC).^54^ A 10 mM stock solution of the surfactant is prepared and subsequently diluted with water to obtain sample concentrations. Sodium dodecyl sulfate (SDS, ≥99.3%, Carl Roth GmbH) is used as received without further purification. All aqueous solutions are prepared using ultrapure water from a Millipore system with a resistivity greater than 18.2 MΩ·cm and a total organic carbon (TOC) content of <5 ppb.

**Figure 1:**
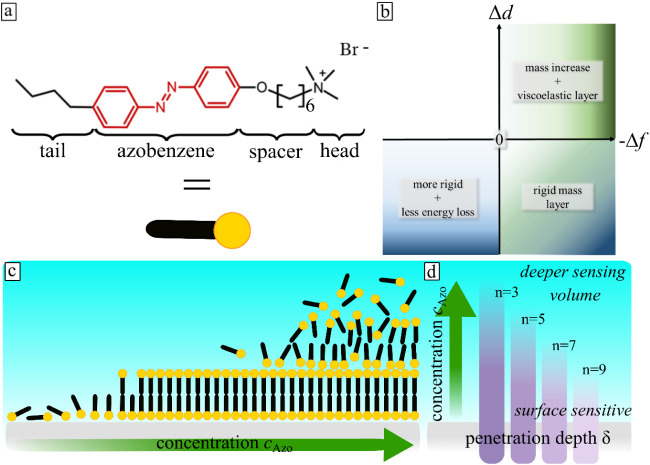
(a) Chemical structure of the azobenzene-based surfactant (AzoC_6_), highlighting the hydrophobic tail, azobenzene unit, spacer, and charged headgroup. (b) Schematic of QCM-D response regimes showing changes in frequency (Δ*f*) and dissipation (Δ*d*) associated with rigid mass loading and viscoelastic layer formation. (c) Conceptual illustration of concentration-dependent adsorption on the QCM-D sensor surface, indicating increasing surface coverage and multilayer formation at higher surfactant concentrations. (d) Schematic representation of shear-wave penetration depth (δ) into the adsorbed layer and surrounding liquid for different odd overtones (*n* = 3, 5, 7, 9). Lower harmonics probe a larger volume extending further into the viscoelastic layer and liquid, while higher harmonics are increasingly surface-sensitive.

### Methods

Quartz crystal microbalance with dissipation monitoring (QCM-D) experiments are conducted using a four-channel Q-Sense E4 instrument (Biolin Scientific). QCM sensors coated with borosilicate (QS-QSX336, LOT Quantum Design GmbH) are employed. All measurements were performed at 23 °C under continuous flow conditions. Sample solutions are introduced into the QCM chamber at a flow rate of 100 μL·min^–1^ using a peristaltic pump (Ismatec IPC ISM935C, Cole-Parmer). Adsorption processes are monitored via the frequency shift (Δ*f*) and dissipation change (Δ*d*) from the third to the ninth overtone,^55^ which provide information on both mass uptake and the viscoelastic properties of the adsorbed layer (Figure 1b). In QCM-D measurements, the sensed mass depends on the penetration depth of the shear wave into the interfacial layer and surrounding liquid. Material that is strongly coupled to the oscillatory shear field contributes primarily to the frequency shift, whereas weakly coupled or highly hydrated regions contribute more strongly to dissipation and are preferentially detected at lower overtones (Figure 1c,d). Prior to each experiment, the resonance frequency of the sensor crystal is measured first in air and then in degassed Millipore water, which is degassed by sonication in an Emmi-H22 (Emag AG) ultrasonic bath for ∼25 min. The temporal evolution of Δ*f* and Δ*d* is recorded until a steady state is reached, defined as the point at which both parameters remain constant for at least 5 min. To prevent air bubble formation during solution exchange, the flow is briefly interrupted.

After each measurement, sensors are cleaned via 10 min of O_2_ plasma treatment (PDC-32G, Harrick Plasma), followed by 30 min of immersion in 2 wt % SDS solution and Millipore water. Crystals were kept wetted until drying under a nitrogen stream and subjected to a second 10 min plasma treatment. Sensors were immediately ready for reuse after cleaning. Successful cleaning was verified by measuring the baseline resonance frequency and dissipation in air and Milli-Q water. Stable baseline values without detectable drift were taken as confirmation of proper sensor cleaning.

### QCM-D Data Analysis: Sauerbrey and Kelvin–Voigt Model

To quantitatively analyze the mechanical properties of the adsorbed *trans*-AzoC_6_ layer, QCM-D data are evaluated using both the Sauerbrey equation and the Kelvin–Voigt viscoelastic model, depending on the rigidity of the adsorbed film. This combined approach enables discrimination between rigid, mass-dominated adsorption regimes and soft viscoelastic layer formation.

### Sauerbrey Analysis of Rigid Adsorbed Layers

For rigid, thin, and laterally homogeneous films with negligible energy dissipation (Δ*d* ≪ 1 × 10^–6^ and overtone-independent frequency shifts), the Sauerbrey equation is applied to estimate the adsorbed mass per unit area:^39,40,50,56^

1 $$\Delta m=-\frac{C\Delta f_{n}}{n}$$

where Δ*m* is the adsorbed mass (ng·cm^–2^), Δ*f*_*n*_ the frequency shift at overtone number *n*, and *C* = 17.7 ng·cm^–2^ Hz^–1^ the Sauerbrey constant for a 5 MHz quartz crystal. Sauerbrey analysis is applied only in concentration regimes exhibiting negligible dissipation changes and harmonic-independent frequency shifts, consistent with rigid coupling of the adsorbed layer to the oscillating crystal.

### Kelvin–Voigt Viscoelastic Model

For surfactant films exhibiting significant dissipation shifts, indicative of viscoelastic behavior, QCM-D data are analyzed using the Kelvin–Voigt viscoelastic model,^39,40,50,57^ as implemented in the Q-Sense/QSoft software. In this framework, the adsorbed layer is treated as a laterally homogeneous, isotropic film of uniform thickness (*h*) and density (ρ_f_), rigidly coupled to the quartz crystal and fully immersed in a Newtonian liquid of known density (ρ_l_) and viscosity (η_l_). The Kelvin–Voigt model represents the adsorbed layer as a parallel combination of an elastic spring and a viscous dashpot, capturing both elastic energy storage and viscous dissipation under oscillatory shear. The complex shear modulus of the film at angular frequency ω_*n*_ = 2π*f*_*n*_ is expressed as

2 $$G^{*}\left(\omega_{n}\right)=G^{\prime }+i\omega_{n}\eta$$

where *G*′ is the shear elastic modulus and η the shear viscosity. Coupling of the oscillating crystal with the viscoelastic layer and surrounding liquid produces shifts in resonance frequency (Δ*f*_*n*_) and dissipation (Δ*d*_*n*_), which are linked to the film’s viscoelastic properties via the standard coupled shear-wave propagation equations and the liquid shear impedance (*Z*_l_). The real and imaginary components of the complex frequency shift correspond to Δ*f*_*n*_ and Δ*d*_*n*_, respectively:

3 $$\Delta f_{n}=-\frac{f_{0}}{n}\frac{h\rho_{f}}{\rho_{q}h_{q}}F\left(G^{\prime },\eta \right)$$

4 $$\Delta d_{n}=\frac{1}{\pi f_{n}}\frac{h\rho_{f}}{\rho_{q}h_{q}}L\left(G^{\prime },\eta \right)$$

where *f*_0_ is the fundamental frequency of the quartz crystal, *f*_*n*_ = *nf*_0_ and *ρ*_f_ and *ρ*_q_ are the densities of the film and quartz, and *h*_q_ is the quartz thickness. The functions *F* and *L* account for elastic energy storage and viscous energy dissipation, respectively.

While the Kelvin–Voigt model provides a convenient effective description of hydrated interfacial films, it is important to note that the fitted parameters (*h*, *G*′, η) are not independent but are strongly coupled through the model equations, which can lead to nonunique solutions, particularly for highly hydrated and vertically stratified layers.^42,58^ In such cases, different combinations of thickness, viscosity, and shear modulus may reproduce the experimental Δ*f*_*n*_ and Δ*d*_*n*_ responses with comparable quality. Therefore, the extracted parameters should be interpreted as effective, model-dependent quantities describing the shear-coupled portion of the film rather than intrinsic material constants. This limitation is especially relevant for soft, water-rich surfactant assemblies, where gradients in composition and viscoelasticity may exist across the film thickness. To assess the robustness of the analysis, we evaluate the sensitivity of the fitted parameters to reasonable variations in input assumptions (e.g., film density *ρ*_f_ and thickness *h*). Thickness variations of ±20% (e.g., due to uncertainty in *ρ*_f_ or hydration level) lead to changes in *G*′ of approximately 30% and in η of approximately 50%. Despite these quantitative variations, the observed trends in viscoelastic behavior (e.g., relative changes between samples or conditions) remain consistent, supporting the reliability of the qualitative conclusions.

Film parameters (*h*, *G*′, and *η*) are determined by simultaneous fitting of Δ*f*_*n*_ and Δ*d*_*n*_ across multiple odd overtones (*n* = 3, 5, 7, 9). This multiovertone fitting leverages the different shear wave penetration depths of the harmonics, enhancing the robustness and internal consistency of the extracted viscoelastic profile. While this approach reduces ambiguity compared to single-overtone analysis, it does not fully eliminate the inherent parameter coupling. The resulting parameters therefore describe the effective viscoelastic response of the adsorbed film and are interpreted as averaged properties of the shear-coupled portion of the layer.

This approach has been widely applied to cationic and anionic surfactant films, polymer layers, and photoresponsive interfacial assemblies, providing quantitative insight into layer thickness, elasticity, viscosity, and vertical stratification of adsorbed materials at solid–liquid interfaces.^48,50,59,60^

## Results and Discussion

To investigate the adsorption behavior of the *trans* isomer of AzoC_6_ (Figure 1a) on solid surfaces, QCM-D experiments are performed on borosilicate substrates at a fixed temperature of 23 °C and a flow rate of 100 μL·min^–1^. The surfactant concentration is systematically varied to probe concentration-dependent adsorption processes.

To facilitate the interpretation of the overtone-dependent QCM-D response, we summarize a conceptual model of the adsorption process and the associated shear-wave penetration depth (δ) in Figure 1c and 1d. Figure 1c depicts the progressive increase in surface coverage and film thickness with increasing surfactant concentration, while Figure 1d schematically illustrates δ for different odd overtones (*n* = 3, 5, 7, 9). Lower harmonics probe a larger volume of the viscoelastic interfacial layer and adjacent liquid, capturing contributions from both the rigidly coupled and hydrated portions of the film. In contrast, higher harmonics are increasingly sensitive to the near-surface region and the mechanically active fraction of the adsorbed layer. This overtone-dependent sensitivity enables the Kelvin–Voigt analysis to resolve the vertical distribution of viscoelastic properties within the adsorbed film. Representative adsorption curves are discussed below to illustrate these harmonic-specific effects.

Figure 2 presents time-resolved QCM-D data at a fixed overtone (*n* = 7), showing frequency shifts (Δ*f*, top panels) and dissipation changes (Δ*d*, bottom panels) during the adsorption of the *trans* isomer of AzoC_6_ at concentrations ranging from 0.005 mM (∼0.01 × CMC) to 2 mM (∼4 × CMC). At the lowest concentration (0.005 mM; Figure 2a, e), Δ*f* exhibits only a minor negative shift of ∼20 Hz, accompanied by negligible dissipation change, indicating the formation of a low-coverage layer of surfactant molecules with minimal viscoelastic contribution. Increasing the concentration to 0.1 mM (∼0.2 × CMC) (Figure 2b,f) produces a more pronounced frequency decrease (Δ*f* ≈ 80 Hz) and a moderate rise in dissipation, suggesting a transition toward more complete surface coverage, possibly involving the formation of a loosely packed or partially hydrated layer. Further increases to 0.5 mM (∼1 × CMC) and 2 mM (∼4 × CMC) (Figure 2c–d,g–h) lead to larger frequency decreases exceeding 150 Hz and concurrent increases in dissipation, particularly at 2 mM where Δ*d* reaches ≈0.2 × 10^–6^. The pronounced frequency reduction reflects substantial mass accumulation, while the enhanced dissipation indicates increased viscoelastic losses associated with the formation of multilayers or diffuse layers, or hydrated aggregates.

**Figure 2:**
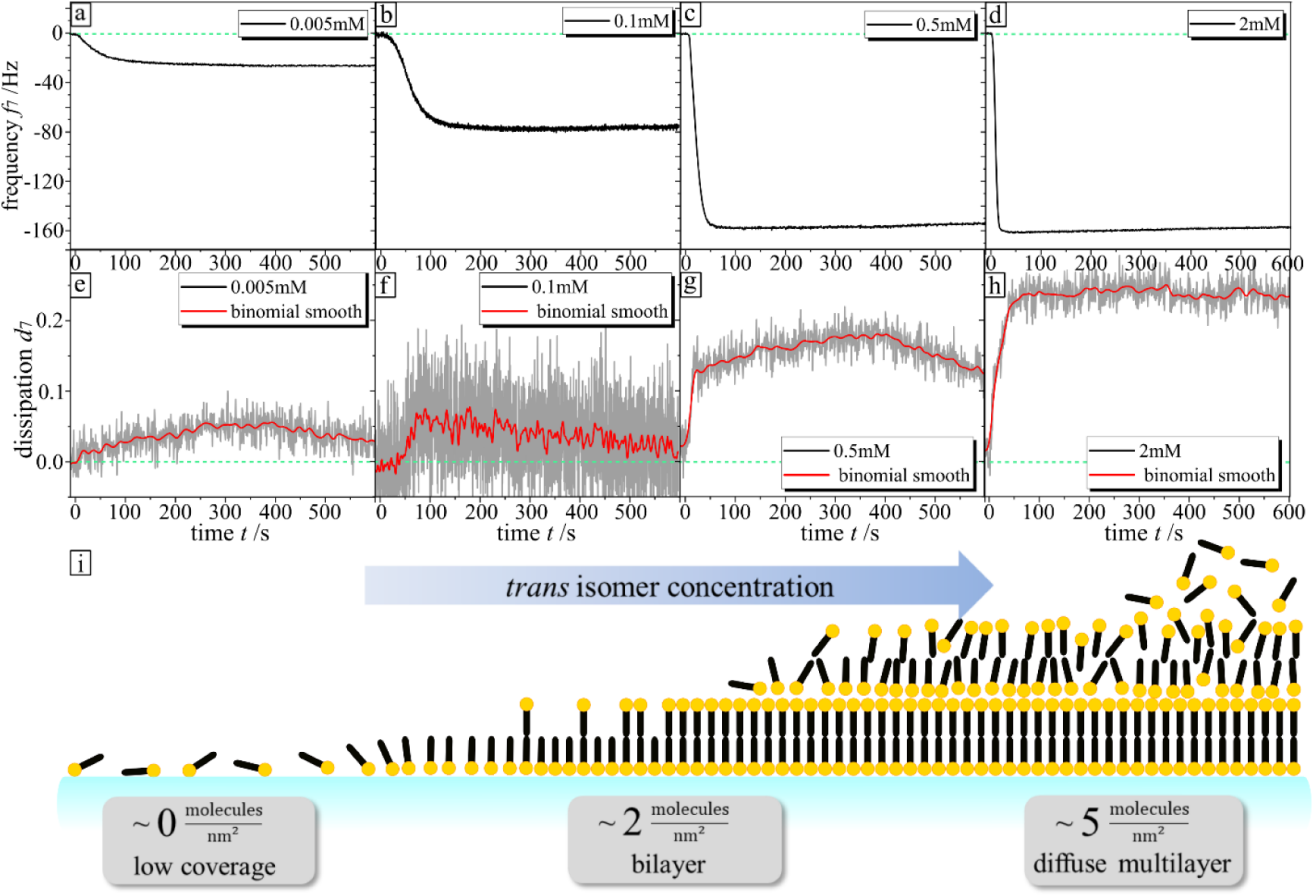
Time-resolved frequency shifts (Δ*f*, top row) and dissipation changes (Δ*d*, bottom row) during adsorption of*trans*-AzoC_6_ at varying concentrations (0.005 mM (∼0.01 × CMC), 0.1 mM (∼0.2 × CMC), 0.5 mM (∼1 × CMC), and 2 mM (∼4 × CMC)). The frequency data (a–d) show a concentration-dependent negative shift, reflecting increasing mass adsorption on the sensor surface. The corresponding dissipation data (e–h) are smoothed using a binomial filter (order = 100) for clarity and reveal an increase in viscoelasticity at higher concentrations (red curves). All measurements are performed under constant flow and temperature conditions. (i) Schematic representation of the adsorbed layer evolution with increasing surfactant concentration, illustrating the transition from a sparse adsorbed layer to a compact bilayer and finally to a diffuse, hydrated multilayer.

These observations are consistent with previous reports on cationic surfactants near and above the CMC, where adsorption beyond the saturation of the initially formed adsorbed layer leads to the buildup of diffuse, solvent-rich multilayers. The schematic in Figure 2i summarizes the evolution of the adsorbed structure with increasing *trans*-isomer concentration: at low coverage, isolated molecules adsorb flat on the surface; at intermediate coverage, a more compact bilayer structure forms; and at high concentrations, a diffuse, hydrated multilayer develops. The combined Δ*f*_7_ and Δ*d*_7_ profiles thus demonstrate a clear concentration-dependent transition from a thin, rigid adsorbed layer to a viscoelastic multilayer of *trans*-AzoC_6_ on borosilicate glass.

A more detailed characterization of the structured layer formation by QCM-D is achieved by analyzing the ratio of Δ*f*_*n*_ to Δ*d*_*n*_ for all recorded overtones. This ratio reflects the viscoelastic properties of the adsorbed layer, as energy dissipation and the acoustic resonance frequency are correlated with the layer’s mechanical behavior. At a given recording time *t*, the ratio can be expressed as

$${layer\;properties∼\frac{\Delta d_{n}\left(t\right)}{-\Delta f_{n}\left(t\right)}\;\;}_{.}$$ 5

This approach highlights the distinct adsorption regimes typically observed in QCM-D (Figure 1b): rigid, mass-dominated films exhibit large negative frequency shifts with low dissipation, whereas soft, viscoelastic layers are characterized by higher dissipation and smaller frequency changes. Layers with higher viscosity or strong hydration display larger Δ*d*_*n*_ values and are located in regime (I), while rigid interfaces appear in regime (II) (Figure 1b).

A key advantage of QCM-D is its simultaneous recording of multiple overtones, where lower overtones probe deeper into the bulk solution and higher overtones are increasingly sensitive to regions near the solid–liquid interface (Figure 1d). Consequently, the response of each overtone provides information about the vertical position within the adsorbed layer: the ninth overtone predominantly reports on material close to the surface, whereas the third overtone reflects properties at higher positions above the interface. This overtone-resolved analysis enables detailed insights into the structural organization and mechanical heterogeneity of the adsorbed film.

With respect to surfactant adsorption, overtone-resolved analysis in Figure 3a–d refines this interpretation by illustrating how the viscoelastic character of AzoC_6_ layers evolves with both concentration and probing depth. At the highest concentration examined (2.5 mM (∼5 × CMC); Figure 3d), dissipation amplitudes decrease with increasing overtone number (*n* = 3, 5, 7, 9), indicating that the outer layers of the adsorbed film are softer and more hydrated, while layers near the surface are comparatively more rigid. Such depth-dependent mechanical gradients are characteristic of soft interfacial assemblies^42^ formed under high surfactant loading.^51,57^

**Figure 3:**
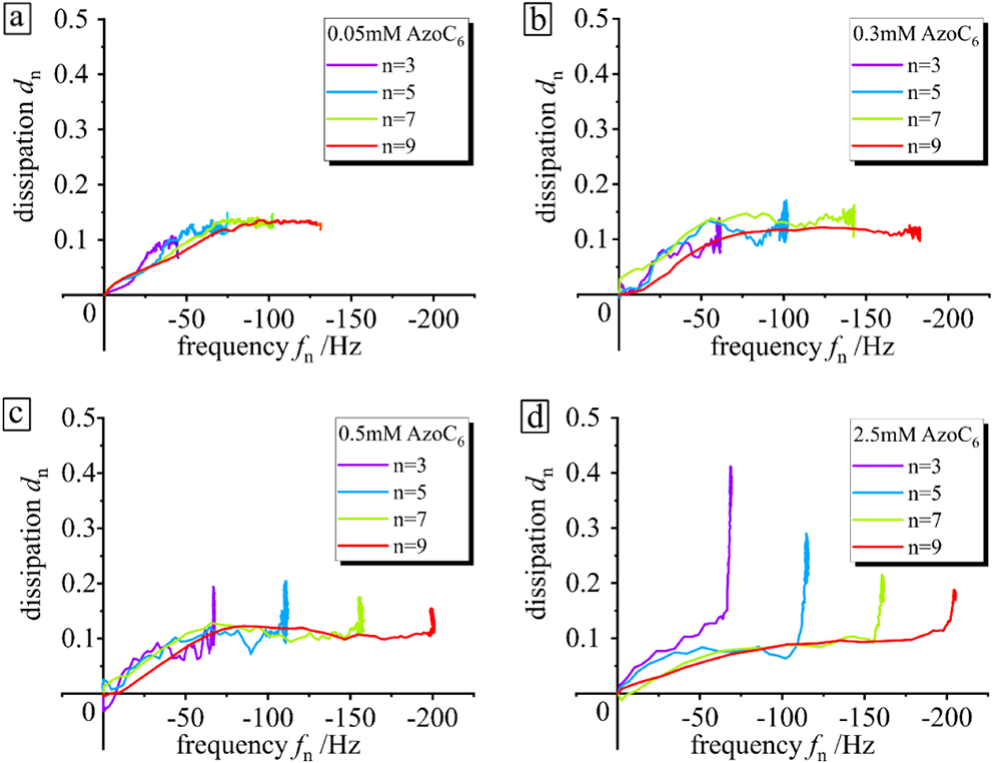
Dissipation versus frequency shifts for different overtones (*n* = 3, 5, 7, 9) at varying AzoC_6_ concentrations: (a) 0.05 mM (∼0.1 × CMC), (b) 0.3 mM (∼0.6 × CMC), (c) 0.5 mM (∼1 × CMC), and (d) 2.5 mM (∼5 × CMC). Increasing concentrations lead to distinct overtone-dependent dissipation-frequency relationships, indicating concentration-dependent changes in viscoelastic film properties.

While Figure 2 captures the overall adsorption behavior, the dissipation–frequency relationships at distinct harmonics provide direct insight into spatial heterogeneity within the interfacial film. At low concentrations (≤0.1 mM (∼0.2 × CMC)), all overtones exhibit nearly uniform responses, consistent with a rigid, thin layer dominated by direct substrate contact. As the concentration increases beyond the CMC (∼0.5 mM (∼1 × CMC)), overtone-dependent differences become pronounced, revealing a viscoelastic gradient within the growing film. Lower harmonics (*n* = 3, 5), which probe deeper regions, show higher dissipation than higher harmonics (*n* = 7, 9), indicating that the near-surface region remains relatively rigid, while the outer layers become softer and more hydrated.

This trend continues at concentrations above 1.5 mM (∼3 × CMC), where the dissipation-frequency slopes broaden and flatten, and are characteristic of a diffuse, solvent-rich multilayer with enhanced energy dissipation. The overtone-dependent separation thus demonstrates that the adsorption of *trans*-AzoC_6_ proceeds via the gradual buildup of a viscoelastic, hydrated interfacial network, rather than by simple compact bilayer formation. These findings emphasize the hierarchical nature of the adsorbed film and highlight the sensitivity of QCM-D to nanoscale structural transitions in light-responsive surfactant systems.

A broader analysis across concentrations (Figure 4a) reveals a gradual change in the viscoelastic response. At low concentrations (≤0.1 mM (∼0.2 × CMC)), adsorption produces rigid, mass-dominated layers with minimal dissipation changes, consistent with sparse surface coverage. Increasing the concentration to 0.5 mM (∼1 × CMC) and above induces a pronounced rise in dissipation relative to the frequency shift, reflecting the formation of viscoelastic, hydrated bilayers and eventually diffuse multilayers. At higher concentrations, the response becomes less clearly systematic, consistent with increased structural heterogeneity of the adsorbed film. Overall, the data indicate a concentration-dependent transition from isolated adsorbed aggregates to extended, hydrated multilayers, governed by the interplay between the electrostatic attraction of the trimethylammonium headgroups and the hydrophobic association of the alkyl tails.

**Figure 4:**
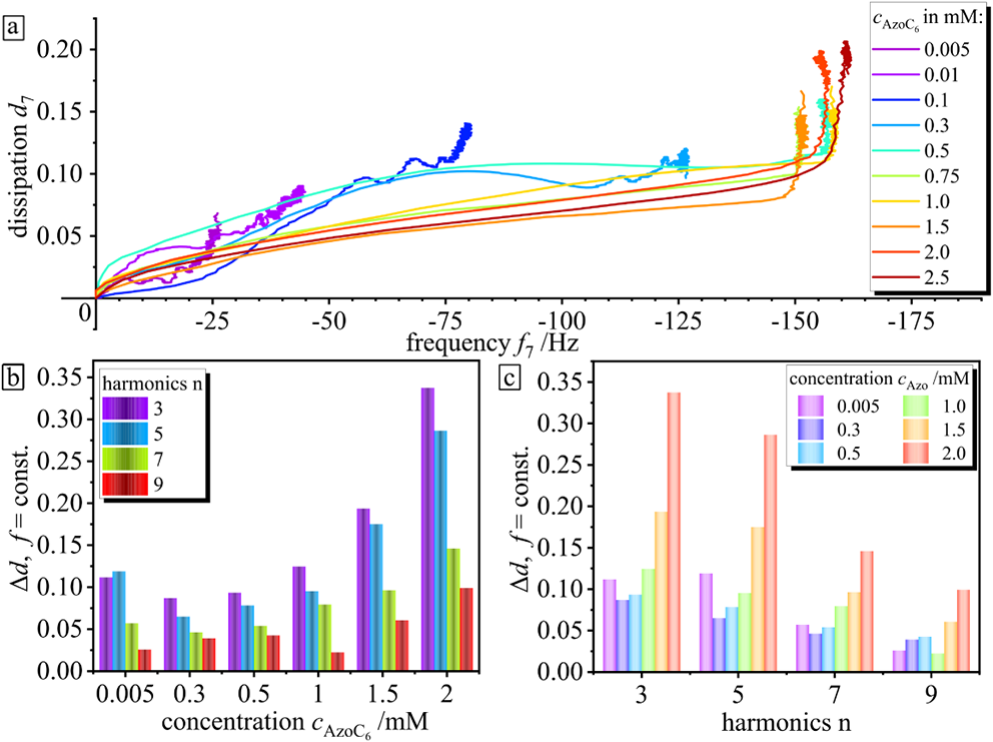
(a) Concentration-dependent dissipation–frequency relationship at the seventh overtone for AzoC_6_ concentrations ranging from 0.005 to 2 mM. (b) Dissipation changes Δ*d* evaluated under conditions where Δ*f* has reached a plateau are plotted versus AzoC_6_ concentration (*c*_AzoC6_) for harmonics *n* = 3, 5, 7, and 9. (c) Corresponding dependence of Δ*d* (*f* = const.) on overtone number for different surfactant concentrations, emphasizing overtone-dependent behavior. An increase in Δ*d* under constant frequency indicates enhanced viscoelastic or structural reorganization within the adsorbed layer without additional mass adsorption. Higher dissipation values at low overtones and high concentrations reflect the formation of thicker, more hydrated, and softer multilayer structures, while lower values correspond to rigid, compact films at low surface coverage.

Once the concentration exceeds the CMC (CMC_*trans*_ = 0.5 mM), dissipation increases markedly, initially for lower harmonics and subsequently for higher overtones. The stronger response at lower harmonics indicates the formation of a softer, more weakly coupled outer region, consistent with multilayer formation. These observations correlate with the overtone-dependent behavior shown in Figure 3 and further support the increasing viscoelastic character of the adsorbed films with the concentration.

To further characterize the mechanical evolution of the adsorbed layers, dissipation changes (Δ*d*) are analyzed under quasi-steady-state conditions, where the frequency shift remains approximately constant (*f* = const.), isolating viscoelastic and structural effects from additional mass adsorption (Figure 4b,c). The same data set is shown in two complementary representations to separately highlight concentration- and overtone-dependent trends. At the lowest concentration (0.005 mM (∼0.01 × CMC)), dissipation remains relatively high despite minimal frequency change, indicating that a small number of AzoC_6_ molecules interact with the surface. These sparsely adsorbed molecules retain significant conformational freedom, resulting in enhanced local viscoelastic losses.

As the concentration increases to 0.3–0.5 mM, Δ*d* decreases, reflecting the formation of a more compact and laterally stabilized layer with reduced internal mobility. At concentrations above 1 mM (∼2 × CMC), dissipation increases again, particularly at low overtones, indicating the formation of thicker, more hydrated, and viscoelastic multilayers. Notably, this increase occurs without further frequency change, confirming that the adsorbed mass remains constant while the film undergoes internal reorganization and partial decoupling from the oscillating surface.

Overall, the concentration-dependent Δ*d* behavior reveals a progression from isolated, mobile molecules to rigid bilayers, and ultimately to dynamic, hydrated multilayer structures, illustrating the hierarchical mechanical organization of *trans*-AzoC_6_ films on borosilicate surfaces.

### Quantitative Characterization of *trans*-AzoC_6_ Adsorption Layers

To quantitatively validate the adsorption regimes inferred from the overtone-resolved frequency and dissipation data, the QCM-D response is analyzed using a combination of Sauerbrey mass evaluation and Kelvin–Voigt viscoelastic modeling. This approach enables a distinction between rigid, mass-dominated adsorption at low surfactant concentrations and viscoelastic multilayer formation at higher concentrations.

### Rigid Adsorption Regime: Sauerbrey Mass Evaluation

At low AzoC_6_ concentrations, dissipation changes remain negligible (Δ*d* ≪ 1 × 10^–6^) and frequency shifts collapse across all recorded overtones, indicating rigid coupling of the adsorbed layer to the oscillating crystal. Under these conditions, the Sauerbrey equation provides a valid estimate of the adsorbed mass per unit area.

Application of the Sauerbrey relation yields adsorbed masses on the order of 50–200 ng·cm^–2^ for concentrations up to 0.1 mM (∼0.2 × CMC),^48^ corresponding to the formation of sparse adsorbed layers or compact regions ranging from single to bilayers with an effective thickness of approximately 1–2 nm. These values are consistent with the qualitative interpretation of the QCM-D data, which indicates rigid, laterally stabilized adsorption at low surface coverage. Importantly, the absence of overtone dependence in both frequency and dissipation confirms that viscoelastic contributions are negligible in this concentration regime.

The molecular length of AzoC_6_ in the *trans* state is approximately 2 nm.^61^ The experimentally determined thickness of ∼1–2 nm at low concentrations is therefore consistent with the adsorption of individual molecules in a partially packed layer. In contrast, the larger thickness values observed near and above the CMC exceed the molecular length and are thus compatible with the formation of bilayer or multilayer assemblies.

### Onset of Viscoelasticity and Kelvin–Voigt Model

At intermediate and high surfactant concentrations, dissipation increases significantly and the frequency response becomes overtone-dependent, signaling a departure from rigid-film behavior. In this regime, mass-only analysis using the Sauerbrey equation underestimates the adsorbed material, necessitating viscoelastic modeling to accurately describe the QCM-D response. Accordingly, the Kelvin–Voigt model is applied to the overtone-resolved data through simultaneous fitting of Δ*f*_*n*_ and Δ*d*_*n*_ for *n* = 3, 5, 7, and 9 (eqs 3 and 4). The extracted parameters such as film thickness (*h*), shear elastic modulus (*G*′), and shear viscosity (*η*) exhibit systematic trends as a function of surfactant concentration. The fitted parameters describe the mechanical response of the shear-coupled portion of the film and should be interpreted as effective, rather than intrinsic, material properties.

The Kelvin–Voigt-derived effective thickness, shear modulus, and viscosity exhibit a pronounced maximum near the CMC (Figure 5). This peak reflects the formation of a mechanically percolated interfacial network in which electrostatically anchored AzoC_6_ headgroups and hydrophobically associated tails form a stress-bearing backbone. At higher concentrations, additional surfactant accumulates predominantly in a diffuse, hydrated overlayer that is only partially coupled to the oscillating shear field. Consequently, the effective mechanical thickness, modulus, and viscosity decrease despite continued mass adsorption, indicating partial mechanical decoupling of the outer layers. Figure 5a depicts that the Sauerbrey thickness increases monotonically and saturates above the CMC, reflecting the continuous accumulation of adsorbed mass. Below the onset of viscoelastic layer formation (≤0.1 mM), the adsorbed films are described by small thicknesses (*h* = 1–2 nm), high elastic moduli (*G*′ ≈ 10^6^–10^7^ Pa), and low viscosities (*η* ≈ 10^–3^ Pa·s), consistent with rigid mono to bilayer structures with limited hydration. As the concentration approaches the CMC (∼0.5 mM), the fitted thickness increases to approximately 3–5 nm, accompanied by a decrease in elastic modulus and a moderate increase in viscosity. This behavior reflects the onset of structural reorganization within the adsorbed layer and enhanced coupling to the surrounding solvent. In contrast, the Kelvin–Voigt thickness exhibits a pronounced maximum close to the CMC, followed by a collapse to a lower plateau. This divergence indicates that, above the CMC, additional surfactant mass accumulates predominantly in a weakly coupled, hydrated overlayer that contributes to the gravimetric signal but not to the mechanical stiffness sensed by the oscillating crystal.

**Figure 5:**
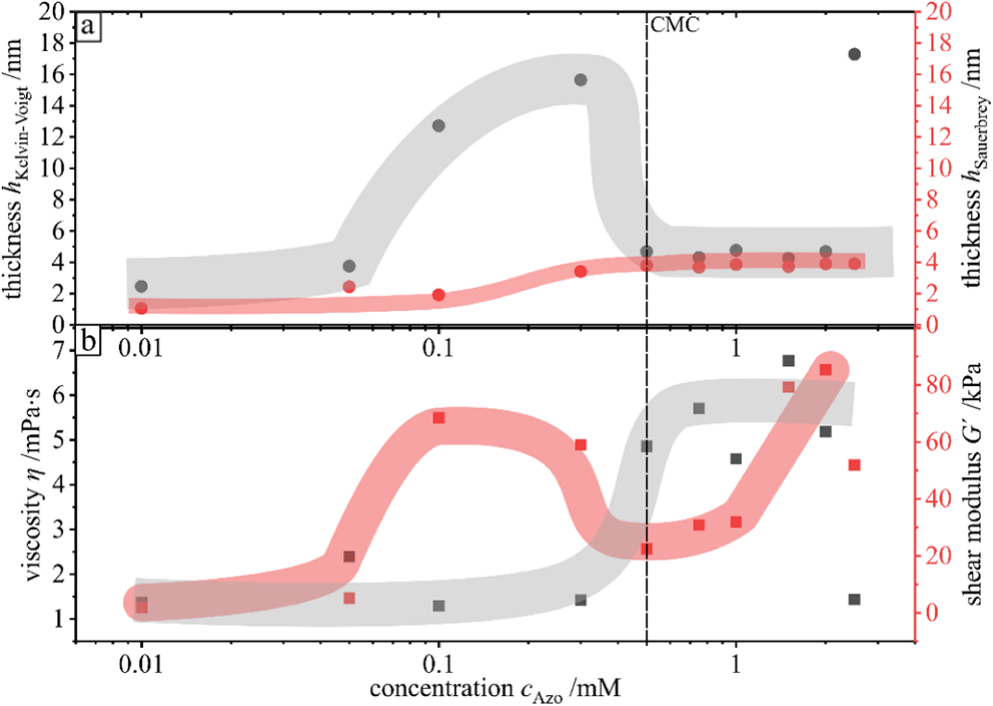
Concentration-dependent evaluation of the mechanical thickness and viscoelastic properties of adsorbed AzoC_6_film on the borosilicate glass surface. (a) Kelvin–Voigt thickness *h*_Kelvin–Voigt_ (black circles) and Sauerbrey thickness *h*_Sauerbrey_ (red circles) as a function of AzoC_6_ concentration. (b) Corresponding shear viscosity η (black squares) and shear modulus *G′* (red squares) extracted from the Kelvin–Voigt model. Both quantities increase as the concentration approaches the CMC, indicating the formation of a mechanically percolated interfacial network. The vertical dashed line marks the critical micelle concentration. Shaded curves are guides to the eye illustrating the characteristic peak–collapse–plateau behavior.

At concentrations above the CMC (≥1 mM), the Kelvin–Voigt model thickness decreases to a lower plateau of ∼4–6 nm, despite the continued increase in the Sauerbrey mass. This divergence demonstrates that additional surfactant accumulates predominantly in a soft, hydrated overlayer that is weakly coupled to the oscillatory shear field. The mechanically active backbone of the film therefore remains limited to a compact interfacial zone of approximately two to three molecular layers, while the outer multilayer contributes mainly to dissipation and gravimetric loading.^39,42,62^ At the CMC, a collapse in *G′* is observed while η remains elevated, indicating a transition from a rigid stress-bearing interfacial network to a more hydrated, dissipative multilayer that is only partially mechanically coupled to the surface. At higher concentrations, *G′* increases again while η approaches a plateau, reflecting progressive densification and partial mechanical recoupling within the multilayer (Figure 5b).

### Vertical Mechanical Stratification of the Adsorbed Film

Although the Kelvin–Voigt model yields a single effective viscoelastic layer, the overtone resolved $\frac{\Delta d}{-\Delta f}$ analysis provides independent evidence for vertical mechanical heterogeneity within the adsorbed AzoC_6_ films (Figure 6). Lower harmonics (*n* = 3, 5) consistently exhibit higher dissipation relative to the frequency shift than higher harmonics (*n* = 7, 9) indicating that the outer regions of the film are softer and more hydrated, while the near-surface region remains mechanically rigid and elastically coupled to the substrate.

**Figure 6:**
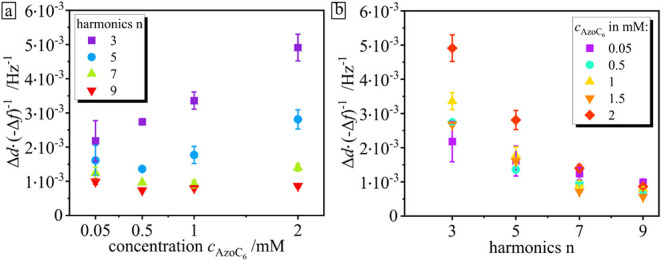
Viscoelastic characterization of AzoC_6_ adsorption on the sensor surface. (a) Normalized acoustic ration $\frac{\Delta d}{-\Delta f}$ as a function of AzoC_6_ concentration (*c*_AzoC6_) for different harmonics (*n* = 3, 5, 7, 9). (b) Dependency of the acoustic ratio on the harmonic order *n* at varying concentrations of AzoC_6_ (0.05 mM to 2 mM). The observed spreading of the harmonics and the magnitude of the $\frac{\Delta d}{-\Delta f}$ ratio indicate the formation of a viscoelastic/soft layer.

This overtone separation becomes more pronounced above the CMC, consistent with the formation of a diffuse, weakly coupled overlayer atop a compact interfacial backbone (see Figure 1c,d). The agreement between Kelvin–Voigt modeling and the model-independent $\frac{\Delta d}{-\Delta f}$ analysis confirms a vertically stratified architecture composed of a mechanically load-bearing inner layer and a solvent-rich outer region.

## Conclusion

We systematically investigate the adsorption of the *trans* isomer of the light-responsive surfactant AzoC_6_ on borosilicate surfaces using QCM-D. Overtone-resolved analysis reveals a concentration-dependent evolution of adsorbed film morphology. At low concentrations (≤0.005 mM, ∼0.01 × CMC), adsorption produces a thin, rigid adsorbed layer with limited surface coverage. Intermediate concentrations (0.1–0.5 mM, ∼(0.2–1) × CMC), below the CMC, favor the formation of compact, laterally stabilized bilayer patches dominated by elastic responses. At higher concentrations (≥1 mM, ∼2 × CMC), dissipation increases markedly, reflecting the formation of hydrated, viscoelastic multilayers.

Kelvin–Voigt modeling quantifies film thickness, shear modulus, and viscosity, showing a peak near the CMC followed by a decrease at higher concentrations as additional surfactant accumulates in a weakly coupled, hydrated outer layer. Model-independent $\frac{\Delta d}{-\Delta f}$ analysis confirms vertical mechanical gradients, with softer, solvent-rich outer layers and more rigid regions near the substrate.

These results demonstrate the hierarchical buildup of AzoC_6_ assemblies, governed by the electrostatic interactions of the head groups and the hydrophobic association of the tails. The combined overtone-resolved QCM-D, Kelvin–Voigt, and $\frac{\Delta d}{-\Delta f}$ analyses provide a quantitative, nanoscale picture of the structural and mechanical organization of photoresponsive surfactant films at the borosilicate glass–water interface.

Although this study focuses on the *trans* isomer, a small fraction of *cis* molecules is expected to be present under static conditions. Previous studies on the same surfactant system reported a *trans*–*cis* ratio at the surface of approximately 80:1 in the dark under comparable conditions [52]. Since the present experiments do not explicitly resolve contributions from the minor *cis* population, its influence on the measured viscoelasticity cannot be fully excluded. However, the adsorption behavior is expected to be dominated by the *trans* isomer. At higher concentrations and under illumination, dynamic *trans*–*cis* photoisomerization coupled with adsorption–desorption kinetics may induce structural changes. The overtone-resolved QCM-D approach used here provides a robust platform for future light-controlled studies.

## Supplementary Material



## Notes

^1^H NMR spectra for azobenzene surfactant (PDF)
