# Supplementary material for: Probing the Depth-Resolved Structure of Adsorbed Azobenzene Surfactant Films by QCM‑D
Source: Langmuir. 2026 Jun 3;42(24):17353–62. doi: 10.1021/acs.langmuir.6c00926 (PMC13296487; doi:10.1021/acs.langmuir.6c00926)
Supplement: Supplementary file 1 [file la6c00926_si_001.pdf]

Supporting information

Probing the Depth-Resolved Structure of Adsorbed Azobenzene Surfactants Film by QCM-D

Maren Umlandt, Philipp Ortner, Nino Lomadze, Marek Bekir, Svetlana Santer,*

Institute of Physics and Astronomy, University of Potsdam, Potsdam, Germany

Section 1. Materiels and Synthesis

$AzoC_6$ was synthetisized following the previously reported procedure. [i] The following reagents were used as received without further purification: 4-butylaniline (95%), sodium nitrite (97%), phenol (≥99%), sodium hydroxide (≥ 97%), sodium carbonate (ChemBeads), hydrochloric acid (37%), acetone (99,7%), potassium carbonate (ChemBeads), 1,6-dibromhexane (96%), chloroform (99,9%), and thrimethylamine solution (31-35 wt.% in ethanol). All chemicals were purchased from Sigma-Aldrich.

^{1}H-NMR characterization of AzoC$_6$

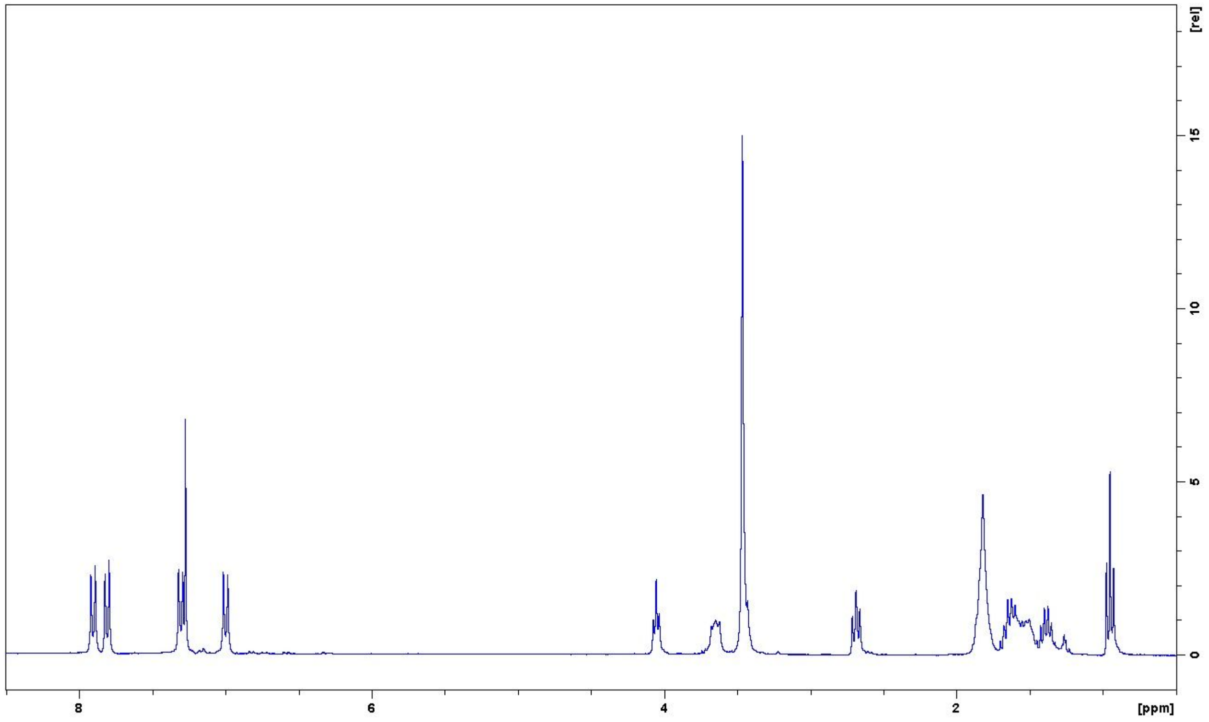

Figure S1 ^{1}H-NMR (CDCl3, ppm): 0.93 (t, J = 7.3 Hz, 3H, CH2CH3), 1.29 - 1.87 (m, 12H, CH2CH2CH2), 2.67 (t, J = 7.7 Hz, 2H, Ph-CH2), 3.44 (s, 9H, NCH3), 3.62 (m, 2H, NCH2), 4.03 (t, J = 6.1 Hz, 2H, PhO-CH2), 6.98 (d, J = 9.0 Hz, 2H, HArom), 7.29 (d, J = 8.4 Hz, 2H, HArom), 7.79 (d, J = 8.3 Hz, 2H, HArom), 7.88 (d, J = 9.0 Hz, 2H, HArom)

[i] Dumont, D.; Galstian, T. V.; Senkow, S.; Ritcey, A. Liguid crystal photoalignment using new photoisomerisable Langmuir-Blodgett films. *Mol. Cryst. Liq. Cryst.* **2002**, *375*, 341– 352, DOI: 10.1080/10587250210555
